# Vaccination with hemagglutinin or neuraminidase DNA protects BALB/c mice against influenza virus infection in presence of maternal antibody

**DOI:** 10.1186/1471-2334-7-118

**Published:** 2007-10-16

**Authors:** Jianjun Chen, Fenghua Zhang, Fang Fang, Haiyan Chang, Ze Chen

**Affiliations:** 1 College of Life Science, Hunan Normal University, Changsha 410081, Hunan, China; 2 State Key Laboratory of Virology, Wuhan Institute of Virology, Chinese Academy of Science, Wuhan 430071, Hubei, China; 3 Shanghai Institute of Biological Products, Shanghai 200052, China

## Abstract

**Background:**

Maternal antibody is the major form of protection against disease in early life; however, its presence interferes with active immunization of offspring. In order to overcome the immunosuppression caused by maternal antibody, several immune strategies were explored in this paper using mouse model and influenza vaccines.

**Results:**

The results showed that: i) when the offspring were immunized with the same vaccine as their mothers, whether inactivated or DNA vaccine, the presence of maternal antibody inhibited offspring immune response and the offspring could not be protected from a lethal influenza virus infection; ii) when the offspring, born to mothers immunized with inactivated vaccine, were immunized with NA DNA vaccine, the interference of maternal antibody were overcome and the offspring could survive a lethal virus challenge; iii) when the offspring were immunized with different DNA vaccine from that for their mothers, the interference of maternal antibody were also overcome. In addition, high-dose inactivated vaccine in maternal immunization caused partial inhibition in offspring when the offspring were immunized with HA DNA vaccine, while lower dose caused no significant immunosuppression.

**Conclusion:**

To avoid the interference of maternal antibody in influenza vaccination of offspring, mothers and their offspring shall not be immunized with the same vaccine. If mothers are immunized with inactivated vaccine, NA DNA vaccine for the offspring shall be effective; and if mothers are immunized with HA (NA) DNA, NA (HA) DNA for the offspring shall be effective.

## Background

Influenza is a highly contagious acute respiratory disease caused by infection of the host respiratory tract with influenza virus [1]. The virus is transmitted in the population of all the age groups, especially in the newborns. The prevention of influenza is currently achieved by subcutaneous injection of inactivated trivalent influenza vaccine. However, because of the immaturity of immune system, effective immune response could not be induced in newborns and therefore vaccination in this age group could not get a satisfactory effect [2,3]. The problem can be solved well by maternal immunization, which can provide the offspring with high titer of maternal antibody [4]. Maternal immunization is beneficial to the offspring, whereas, it also brings the problem of immunosuppression. The presence of the maternal antibody inhibits the offspring immune response to specific antigen [5-7], and the long-lasting immunosuppression often delays the vaccination of the offspring. In young infants, a period susceptible to influenza exists when the maternal antibody titer is too low to provide immunoprotection but is enough to inhibit the active immune response to vaccines. Thus, it is necessary to develop an effective immune strategy to overcome the immunosuppression caused by maternal antibody.

In this paper, we explored the interference of maternal antibody in offspring immune response to influenza vaccines as well as the immune strategy to overcome the interference. We found that, when the offspring were immunized with the same vaccine as their mothers, whether inactivated or DNA vaccine, the active immune response in offspring would be inhibited by the presence of maternal antibody. However, the interference could be overcome under the following situations: immunization of offspring with Neuraminidase (NA) DNA vaccine when the mother were once immunized with inactivated vaccine, or immunization of offspring with different DNA vaccine from that for their mothers, i.e. Hemagglutinin (HA) DNA or NA DNA. These results provided an experimental basis for overcoming the immunosuppression caused by maternal antibody in clinic.

## Results

### Maternal immunization with inactivated vaccine inhibited the effect of inactivated vaccine on protection of offspring

In order to explore whether maternal immunization with inactivated influenza vaccine interferes with the immune effect of inactivated influenza vaccine in offspring, we performed the following test. The female BALB/c mice aged 6–8 weeks were divided into seven groups. In control group, all the mice were unimmunized, including their natal offspring. In three of the six experimental groups, the female mice were unimmunized, but their offspring were immunized with 1.0 μg, 0.1 μg and 0.01 μg of inactivated influenza virus A/PR/8/34 vaccine at age of 1 week respectively, and boosted 3 weeks later with the vaccine at the same dose as primed. In other three experimental groups, the female mice were immunized twice, 3 weeks apart, with 1.0 μg, 0.1 μg and 0.01 μg of inactivated influenza vaccines respectively, and the offspring were immunized twice at age of 1 and 4 weeks respectively, with the same vaccine at the same doses as those for their mothers. The sera of offspring were collected by tail vein bleeding 3 weeks after primary immunization and 1 week after booster respectively and determined for IgG antibody titer by ELISA. As shown in Table 1, when the mothers were immunized with 1.0 μg and 0.1 μg of inactivated vaccine, the antibody titers in their offspring after booster were lower than those after the primary immunization. However, when the female mice were immunized with 0.01 μg of inactivated vaccine, the antibody titers in their offspring after booster were slightly higher. In contrast, the offspring of the unimmunized mothers had higher antibody levels after booster than after the primary immunization.

**Table 1:** Influence of maternal antibodies on protective effect of offspring immunized with the same inactivated vaccine as their mothers^a^

Dose (μg)		Serum IgG titers^b ^in offspring ELISA (2^n^)^c^		Lung virus titers^b ^(log_10 _TCID_50_)	Survival offspring/Tested offspring (3 weeks)
			
Female mice	Offspring	21 days after primary immunization	7 days after booster		
1.00	1.00	14.8 ± 0.50	13.3 ± 0.30	3.7±0.00	0/7
0.10	0.10	13.8 ± 0.90	11.5 ± 0.60	4.7 ± 0.23	0/7
0.01	0.01	9.7 ± 0.60	11.3 ± 0.50	4.8 ± 0.71	0/7
Unimmunized	1.00	14.3 ± 0.60	16.7 ± 0.60	ND*	7/7*
Unimmunized	0.10	12.7 ± 0.60	16.0 ± 1.00	3.4 ± 0.54*	6/6*
Unimmunized	0.01	11.3 ± 0.50	13.0 ± 0.00	3.4 ± 0.54*	5/6*
Unimmunized	Unimmunized	<1	<1	5.4 ± 0.10	0/7

^a ^The female mice were immunized twice, 3 weeks apart, with various doses of inactivated vaccine. The offspring were immunized at ages of 1 and 4 weeks, respectively, with the same vaccine as their mothers. Serum samples from offspring were collected 3 weeks after primary immunization and 1 week after booster. The serum antibody titers were measured by ELISA. One week after booster, the offspring were challenged with a lethal dose of A/PR/8/34 (20 × LD_50_). Lungs were taken out from at least three mice in each group 3 days after challenge for virus titration by standard MDCK assay. Survival rates of mice were measured 3 weeks after challenge.

^b ^Values represent mean ± S.D. of each group.

^c ^The serum samples were diluted 2-fold serially and "n" represents the dilution factor.

* Significant difference (*p *< 0.05). ND: virus not detected

**Figure 1:**
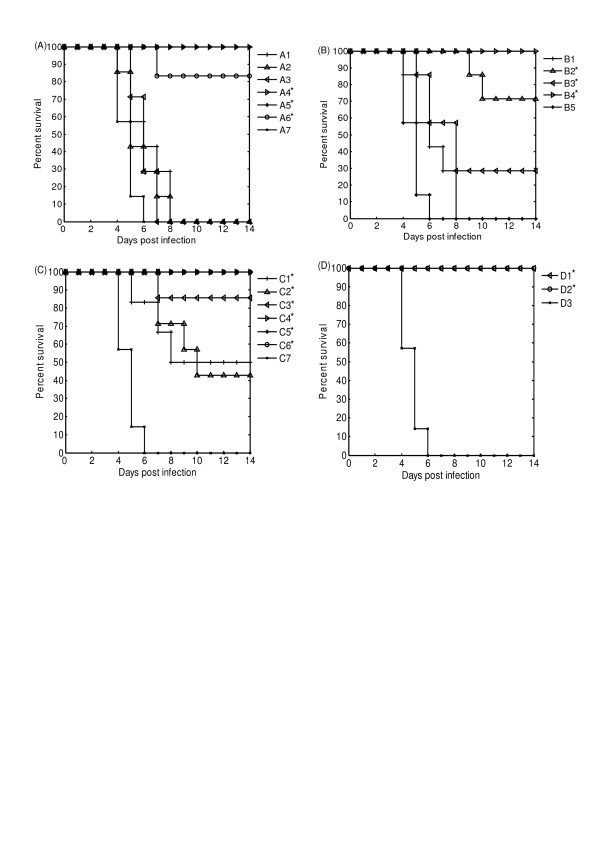
Survival of offspring after lethal A/PR8/34 virus challenge. The offspring were vaccinated as described in result section. One week after second immunization, the offspring were challenged with a lethal dose of A/PR/8/34 (20 × LD_50_). Survival rates of mice were measured after viral challenge. Panel A: (A1) Immunization of both mothers and the offspring with 1.0 μg of inactivated vaccine; (A2) Immunization of both mothers and the offspring with 0.1 μg of inactivated vaccine; (A3) Immunization of both mothers and the offspring with 0.01 μg of inactivated vaccine; (A4) Immunization of offspring born to unimmunized mothers with 1.0 μg of inactivated vaccine; (A5) Immunization of offspring born to unimmunized mothers with 0.1 μg of inactivated vaccine; (A6) Immunization of offspring born to unimmunized mothers with 0.01 μg of inactivated vaccine; (A7) Negative control. Panel B: (B1)Immunization of both mothers and the offspring with 30 μg of HA DNA; (B2) Immunization of offspring born to unimmunized mothers with 30 μg of HA DNA; (B3) Immunization of both mothers and the offspring with 30 μg of NA DNA; (B4) Immunization of offspring born to unimmunized mothers with 30 μg of NA DNA; (B5) Negative control. Panel C: (C1) Immunization of mothers with1.0 μg of inactivated vaccine and the offspring with 30 μg of HA DNA; (C2) Immunization of mothers with 0.1 μg of inactivated vaccine and the offspring with 30 μg of HA DNA vaccine; (C3) Immunization of mothers with 0.01 μg of inactivated vaccine and the offspring with 30 μg of HA DNA; (C4) Immunization of mothers with 1.0 μg of inactivated vaccine and the offspring with 30 μg of NA DNA; (C5) Immunization of mothers with 0.1 μg of inactivated vaccine and the offspring with 30 μg of NA DNA; (C6)Immunization of mothers with 0.01 μg of inactivated vaccine and the offspring with 30 μg of NA DNA; (C7) Negative control. Panel D: (D1) Immunization of mothers with 30 μg of HA DNA and the offspring with 30 μg of NA DNA; (D2) Immunization of mothers with 30 μg of NA DNA and the offspring with 30 μg of HA DNA; (D3) Negative control. *Significant differences (*p *< 0.05) compared to negative controls as determined by Log Rank test.

All the offspring in experimental groups were challenged with a lethal dose (20 × LD_50_) of influenza virus strain A/PR/8/34 by intranasal drip 1 week after the booster. At least three offspring in each group were dissected 3 days after challenge, and their lungs were taken out for virus titration. The rest offspring were observed for 3 weeks to calculate the survival rate. As shown in Table 1, when both the mothers and their offspring were immunized twice with inactivated influenza vaccine, regardless of the doses, all the offspring showed high lung virus titer and died within 2 weeks after challenge. However, when the female mice were unimmunized, their offspring immunized with 1.0 μg, 0.1 μg and 0.01 μg of inactivated influenza vaccine had the survival rates of 100%, 100% and 83.3% respectively (Table 1 and Figure 1A), with significantly lower lung virus titers than offspring in control group. All offspring in control group died within 7 days after challenge, and their lung virus titers were the highest among the seven groups.

The above-mentioned results indicated that the maternal immunization with inactivated vaccine inhibited the immune response induced by the same vaccine in offspring.

### Maternal immunization with DNA vaccine inhibited the effect of the same DNA vaccine on protection of offspring

In order to explore whether the maternal immunization with DNA inhibits the immune effect of the same vaccine in offspring, we performed the following test. The female BALB/c mice aged 6–8 weeks were divided into five groups. In control group, both the female mice and their offspring were unimmunized. In two of the four experimental groups, the female mice were unimmunized, but their offspring were immunized at age of 1 week with 30 μg of HA DNA and 30 μg of NA DNA, respectively, and boosted 3 weeks later with the same DNA vaccine at the same dose as primed. In other two experimental groups, the female mice were immunized twice (at a 3-week interval) with 30 μg of HA DNA and 30 μg of NA DNA, respectively, and the offspring were immunized twice at ages of 1 and 4 weeks respectively, with the same DNA vaccine at the same dose as that for their mothers.

The sera of offspring were collected by tail vein bleeding both 3 weeks after primary immunization and 1 week after booster and determined for HA or NA antibody titer. As shown in Table 2, when both the mothers and their offspring were immunized with HA DNA, the HA antibody titers in offspring after booster were lower either than those after primary immunization or than those of offspring born to unimmunized mothers. Similarly, when the mothers and their offspring were immunized with NA DNA, the NA antibody titers of offspring after booster were also lower either than those after primary immunization or than those of offspring born to unimmunized mothers. In contrast, offspring born to unimmunized mothers had higher antibody titers after booster than those after primary immunization with either HA or NA DNA.

**Table 2:** Influence of maternal antibodies on protective effect of offspring immunized with the same DNA vaccine as that for their mothers^a^

		Serum IgG titers^b ^in offspring					
				
Plasmid		ELISA (2^n^)^c^		NI assay (2^n^)^c^		Lung virus titers^b ^(log_10 _TCID_50_)	Survival offspring/Tested offspring (3 weeks)
			
Plasmid for female mice	Plasmid for offspring	21 days after primary immunization	7 days after booster	21 days after primary immunization	7 days after booster		
30 μg HA	30 μg HA	15.0 ± 0.00	13.3 ± 0.60			4.3 ± 0.35	0/7
Unimmunized	30 μg HA	11.3 ± 0.50	14.3 ± 0.50			4.0 ± 0.58*	5/7*
30 μg NA	30 μg NA			5.6 ± 0.90	3.3 ± 0.60	4.0 ± 0.70	2/7
Unimmunized	30 μg NA			4.6 ± 0.60	8.5 ± 0.70	2.8 ± 0.35*	7/7*
Unimmunized	Unimmunized	<1	<1	<3	<3	5.7 ± 0.00	0/7

^a ^Female mice were immunized twice, 3 weeks apart, with 30 μg HA DNA or 30 μg NA DNA. The offspring were immunized at ages of 1 and 4 weeks, respectively, with the same vaccine as their mothers. Serum samples from offspring were collected 3 weeks after primary immunization and 1 week after booster. The anti-HA antibody titers were measured by ELISA. The anti-NA antibody titers were measured by NI assay. One week after booster, the offspring were challenged with a lethal dose of A/PR/8/34 (20 × LD_50_). Lungs were taken out from at least three mice in each group 3 days after challenge for virus titration by standard MDCK assay. Survival rates of mice were measured 3 weeks after challenge.

^b ^Values represent mean ± S.D. of each group.

^c ^The serum samples were diluted 2-fold serially and "n" represents the dilution factor.

*Significant difference (*p *< 0.05)

The offspring in all groups were challenged to evaluate the immune protection by lung virus titers and survival rates, as described above. As shown in Table 2, when both the female mice and their offspring were immunized with HA DNA, the survival rate of offspring was 0%; and when both with NA DNA, the survival rate of offspring was 28.6%. However, when the female mice were unimmunized, the offspring immunized with HA DNA and NA DNA showed the survival rates of 71.4% and 100%, respectively (Table 2 and Figure 1B), and they had significantly lower lung virus titers than the offspring in control group, and lower titers than those born to immunized mothers as well.

The above-mentioned results indicated that the maternal antibody induced by DNA vaccine inhibited the immune effect of the same DNA vaccine on protection of offspring.

### Maternal immunization with inactivated vaccine partially inhibited the effect of HA DNA vaccine on protection of offspring

The female BALB/c mice aged 6–8 weeks were divided into four groups. In control group, both the female mice and their offspring were unimmunized. In the three experimental groups, the female mice were immunized twice, 3 weeks apart, with 1.0 μg, 0.1 μg and 0.01 μg of inactivated influenza vaccine respectively, and their offspring were immunized with 30 μg of HA DNA at age of 1 week and boosted 3 weeks later.

The sera of offspring were collected by tail vein bleeding 3 weeks after primary immunization and 1 week after booster respectively and determined for HA antibody titer by ELISA. As shown in Table 3, when the mothers were immunized with 1.0 μg of inactivated vaccine, the antibody titer in offspring after booster was lower than that after primary immunization. When the mothers were immunized with 0.1 μg and 0.01 μg of inactivated vaccine, the antibody titer in offspring after booster were only slightly higher than those after primary immunization. The offspring in all groups were challenged with a lethal dose of influenza virus as described above. As shown in Table 3, when the female mice were immunized with 1.0 μg and 0.1 μg of inactivated vaccine and their offspring with HA DNA, the protective rates of offspring were 50% and 42.8% respectively (*p *> 0.05). However, when the female mice were immunized with 0.01 μg of inactivated vaccine, the protective rate of offspring was 85.7% (*p *< 0.05) (Table 3 and Figure 1C). The offspring in the three experimental groups had significantly lower lung virus titers than control mice, and the offspring born to the mothers immunized with 0.01 μg of inactivated vaccine had the lowest titer among the three groups. The results indicated the dose-dependent inhibition of maternal immunization with inactivated vaccine on immunization of offspring with HA DNA.

**Table 3:** Protection against a lethal influenza virus challenge in offspring immunized with DNA vaccine born to the mothers immunized with inactivated vaccine^a^

		Serum IgG titers^b ^in offspring					
				
Dose (μg) of Inactivated vaccine for female mice	Plasmid for offspring	ELISA (2^n^)^c^		NI assay (2^n^)^c^		Lung virus titers^b ^(log_10 _TCID_50_)	Survival offspring/Tested offspring (3 weeks)
				
		21 days after primary immunization	7 days after booster	21 days after primary immunization	7 days after booster		
1.00	30 μg HA	14.8 ± 0.50	13.3 ± 0.30			3.3 ± 0.35*	3/6
0.10	30 μg HA	11.7 ± 0.50	12.5 ± 0.60			3.8 ± 0.23*	3/7
0.01	30 μg HA	11.3 ± 0.60	13.0 ± 0.00			3.0 ± 0.71*	6/7*
1.00	30 μg NA			4.3 ± 0.60	7.0 ± 1.00	1.9 ± 0.58*	7/7*
0.10	30 μg NA			3.7 ± 0.60	7.2 ± 0.80	1.5 ± 0.24*	7/7*
0.01	30 μg NA			4.3 ± 0.60	8.0 ± 1.00	0.9 ± 0.83*	6/6*
Unimmunized	Unimmunized	<1	<1	<3	<3	5.3 ± 0.35	0/7

^a ^Female mice were immunized twice, 3 weeks apart, with various doses of inactivated vaccine. The offspring were immunized with 30 μg HA or 30 μg NA at ages of 1 and 4 weeks respectively. Serum samples from offspring were collected 3 weeks after primary immunization and 1 week after booster. The anti-HA antibody titers were measured by ELISA. The anti-NA antibody titers were measured by NI assay. One week after booster, the offspring were challenged with a lethal dose of A/PR/8/34 (20 × LD_50_). Lungs were taken out from at least three mice in each group 3 days after challenge for virus titration by standard MDCK assay. Survival rates of mice were measured 3 weeks after challenge.

^b ^Values represent mean ± S.D. of each group.

^c ^The serum samples were diluted 2-fold serially and "n" represents the dilution factor.

*Significant difference (*p *< 0.05)

### Maternal immunization with inactivated vaccine could not inhibit the effect of NA DNA vaccine on protection of offspring

The BALB/c mice aged 6–8 weeks were divided into four groups. In control group, both the female mice and their offspring were unimmunized. In three experimental groups, the female mice were immunized twice, 3 weeks apart, with 1.0 μg, 0.1 μg and 0.01 μg of inactivated influenza vaccine respectively, and their offspring were immunized with 30 μg of NA DNA at age of 1 week and boosted 3 weeks later.

The sera of offspring were collected by tail vein bleeding both 3 weeks after primary immunization and 1 week after booster and determined for NA antibody titer by NI assay. As shown in Table 3, the NA antibody titers of offspring after booster were much higher than those after primary immunization. The offspring in experimental groups were challenged with a lethal dose of influenza virus as described above. After immunized with NA DNA, all the offspring born to the immunized mothers showed a survival rate of 100% (Table 3 and Figure 1C), and correspondingly, their lung virus titers were far lower than that in control group. The results showed no influence of maternal immunization with inactivated vaccine on immunization of offspring with NA DNA.

### Maternal immunization with one kind of DNA vaccine could not inhibit the effect of another kind of DNA vaccine on protection of offspring

In order to explore whether immunization of female mice and their offspring with different DNA vaccines could overcome the inhibition of maternal antibody, we performed the following test. The BALB/c mice aged 6–8 weeks were divided into three groups. In control group, both the female mice and their offspring were unimmunized. In one experimental group, the female mice were immunized twice, 3 weeks apart, with 30 μg of HA DNA, and their offspring were immunized with 30 μg of NA DNA at age of 1 week and boosted 3 weeks later. In another experimental group, the female mice were immunized twice, 3 weeks apart, with 30 μg of NA DNA, and their offspring were immunized with 30 μg of HA DNA at age of 1 week and boosted 3 weeks later.

The sera of offspring were collected by tail vein bleeding both 3 weeks after primary immunization and 1 week after booster and determined for HA or NA antibody titer. As shown in Table 4, when the mothers were immunized with NA DNA, and their offspring with HA DNA, the HA antibody titers of offspring after booster were higher than those after primary immunization. Similarly, when the mothers were immunized with HA DNA, and their offspring with NA DNA, the NA antibody titers of offspring after booster were higher than those after primary immunization. The offspring were challenged with a lethal dose of influenza virus as described above. As shown in Table 4, all the survival rates of offspring in the two experimental groups were 100% (also shown in Figure 1D), and correspondingly, their lung virus titers were significantly lower than that of offspring in blank control group. It indicated that the immunization of female mice and their offspring with different DNA vaccines could overcome the interference of maternal antibody in offspring.

**Table 4:** Protection against a lethal influenza virus challenge in offspring immunized with different DNA vaccine from that for their mothers ^a^

		Serum IgG titers^b ^in offspring					
				
Plasmid for female mice	Plasmid for offspring	ELISA (2^n^)^c^		NI assay (2^n^)^c^		Lung virus titers^b ^(log_10 _TCID_50_)	Survival offspring/Tested offspring (3 weeks)
				
		21 days after primary immunization	7 days after booster	21 days after primary immunization	7 days after booster		
30 μg HA	30 μg NA			4.3 ± 0.60	7.0 ± 1.40	1.2 ± 0.57*	7/7*
30 μg NA	30 μg HA	12.0 ± 0.60	15.7 ± 0.60			3.8 ± 0.23*	7/7*
Unimmunized	Unimmunized	<1	<1	<3	<3	5.5 ± 0.16	0/7

^a ^Female mice were immunized twice, 3 weeks apart, with 30 μg HA DNA or 30 μg NA DNA. The offspring were immunized with the different vaccines from that for their mothers at ages of 1 and 4 weeks respectively. Serum samples from offspring were collected 3 weeks after primary immunization and 1 week after booster. The anti-HA antibody titers were measured by ELISA. The anti-NA antibody titers were measured by NI assay. One week after booster, the offspring were challenged with a lethal dose of A/PR/8/34 (20 × LD_50_). Lungs were taken out from at least three mice in each group 3 days after challenge for virus titration by standard MDCK assay. Survival rates of mice were measured 3 weeks after challenge.

^b ^Values represent mean ± S.D. of each group.

^c ^The serum samples were diluted 2-fold serially and "n" represents the dilution factor.

*Significant difference (*p *< 0.05)

## Discussion

Maternal immunization is the major form of protection against many infectious diseases in early life, since IgG Ab can transfer from pregnant women to their foetus through placenta, colostrums and milk [8-11]. Both pregnant women and young infants were found to be vulnerable to serious sequelae of influenza infection [4]. Vaccination in pregnant women is beneficial to both mother and infant. It has been shown that, after women in the last trimester of pregnancy were given trivalent inactivated influenza virus vaccine, high IgG antibody titers to maternal vaccine antigens were detected in cord and infant serum [12]. The passive antibodies given by mother could delay the onset and decrease the severity of influenza disease in young infants [13]. Inactivated influenza vaccine is currently recommended by the US CDC (Centers for Disease Control and Prevention in Atlanta, Georgia, USA) for all pregnant women, especially those in the second or third trimester during influenza seasons or those with high risk conditions [14]. Besides inactivated vaccine, we have found that immunization of mothers with plasmid DNAs encoding influenza HA or NA gene could afford protection to the offspring from a lethal viral infection [15].

On the other hand, the presence of maternal antibody can inhibit the active immune response of offspring. The titer of maternal antibody decreases gradually with the growth of offspring, but even low titer can interfere with the active immune response in offspring [16]. Van Maanen *et al*. [7] have studied the influence of maternal antibody induced by equine influenza vaccine on the immune effect of the same vaccine in foals. The result showed that the maternal antibody interfered with the vaccination of influenza vaccine in foals aged 24–28 weeks. Radu *et al*. [6] have discovered that the immunization of female mice with inactivated influenza virus WSN (A/WSN/32, H1N1) vaccine inhibited the immune effect of the same vaccine in their offspring. Apart from influenza vaccine, the presence of maternal antibody also showed influence on immunization of offspring with many other vaccines. Albrecht *et al*. [17] have investigated the influence of maternal antibody on the vaccination of live attenuated measles vaccine in 34 infants aged 12 months. The seroconversion of measles antibody was not observed in the children with high maternal antibody titers but observed in the children with lower maternal antibody titers, though the induced antibody titers were significantly lower than those of children without maternal antibody. Letson *et al*. [18] have found that, when immunized with inactivated hepatitis A virus (HAV) vaccine within one year old, the maternal antibody-positive children had the significantly lower HAV antibody titer than the maternal antibody-negative children. The passively acquired antibody has also been proved a major interfering factor of immunization with respiratory syncytial virus (RSV) vaccine in offspring. In the study conducted by Murphy *et al*. [19], when cotton rats were injected with the antisera against RSV, and then immunized with the recombinant poxvirus expressing the F (fusion) and G (large) proteins of RSV, the injected antisera inhibited production of the F and G antibodies induced by recombinant poxvirus vaccine. It is thus clear that, though maternal antibody can protect offspring against infectious disease, it can inhibit the active immune response in offspring. Both the protective and inhibitory effects diminish gradually with the growth of offspring. This diminishing period is the infection-susceptible period for offspring.

In our study, we also explored the inhibition of maternal antibody on the immunization of offspring. The influence of maternal antibody on the immunization of offspring was studied by determination of antibody titer and by virus challenge test. The immunosuppression was observed when the mothers and their offspring were immunized with the same vaccines, either inactivated or DNA vaccines. Few or even no offspring survived the lethal virus challenge (see Table 1 and 2). The result indicated that immunization of female mice and their natal offspring with the same vaccine may bring strong inhibition on offspring immune response.

Ways have been looking for to deal with the interference of maternal antibody. The first one is by vaccinating the offspring when maternal antibody declines to a certain extent [20], or by giving the primary immunization before the disappearance of maternal antibody and booster after its disappearance [21]. However, the delayed immunization of offspring may increase the opportunity of infection. The second one is by shortening the interval between primary and booster immunizations of offspring. For example, as reported by Harte *et al*. [22], when the offspring were immunized with formalin-fixed malaria vaccine and boosted only 10 days later, the interference of maternal antibody was overcome. Yet it is not known whether the immune strategy is suitable for other vaccines. The third way is by giving high-dose vaccines for offspring, but this may cause the increase of adverse reactions or even the occurrence of death. Knudsen *et al*. [23] found that the mortality rate of children in Western Africa immunized with high dose of live attenuated measles vaccine was significantly higher than that of the children with standard dose of the vaccine. The fourth way is by immunizing mothers and their offspring with vaccines of different forms. Capozzo *et al*. [24] reported that it was workable to vaccinate female mice with the DNA vaccine pTET1pp encoding tetanus toxin fragment C (Frag C), and their offspring with the Samonella vector carrying plasmid pTET1pp by nasal drip. The antibody titers in offspring after booster were comparable to those of maternal antibody-negative neonatal mice immunized with the same vaccine. Schlereth *et al*. [25] found that the immunization of cotton rats with the recombinant vesicular stomatitis virus (VSV) expressing HA gene of measles virus by nasal drip induced neutralizing antibody which could protect the animals even in the presence of antisera induced by live attenuated measles vaccine.

DNA vaccine has also been tested in search of ways to overcome the interference of maternal antibody. Manickan *et al*. [26] found that the maternal antibody induced by primary immunization with live herpes simplex virus (HSV) vaccine and booster with inactivated HSV vaccine interfered with the immunization of offspring with inactivated HSV vaccine, however, no interference was observed when offspring were immunized with plasmid DNA encoding glycoprotein B (gB). Premenko-Lanier *et al*. [27] once injected infant rhesus monkeys with the antisera induced by the infection with measles virus, then immunized with mixed DNA vaccines containing the hemagglutinin (HA), fusion protein (F) and nucleoprotein (NP) of measles virus. The result showed that the immunization with mixed DNA vaccines could overcome the interference of maternal antibody on immune response. However, different results have also been observed in other studies. Wang *et al*. [28] reported that the passive immunization of offspring with antisera, separated from their mothers immunized with inactivated rabies vaccine, inhibited the immune response of rabies virus glycoprotein (gp) DNA vaccine. This might be largely due to the low immunogenicity of rabies DNA vaccine. High immunogenicity of vaccine is an important factor in overcoming the interference of maternal antibody [24]. Besides this, Pertmer *et al*. [29] found that the maternal antibody induced by a sublethal influenza virus infection inhibited the generation of antibody after immunization of offspring with HA DNA, but did not inhibit the generation of antibody after immunization with NP DNA. The different effects were thought to be as a result of different sites at which the two kinds of DNA vaccines were expressed. The HA protein was expressed on the cell membrane and the NP in the cell. The maternal antibody could interfere with the IgG antibody induced by membrane protein but could not interfere that induced by intracellular protein.

Several mechanisms have been suggested as mediating the inhibitory influence of maternal antibodies on infant responses. But it essentially depends upon the maternal antibodies-vaccine antigen ratio at the time of immunization. The inhibition of infant responses will not occur if maternal antibodies do not persist until completion of the infant immunization schedule, as is the case for most current vaccines [30]. In this paper, we have tried several immune strategies to overcome the interference of maternal antibody with immunization of offspring. When the mothers were immunized with inactivated vaccine and their offspring with DNA vaccine, 1.0 μg and 0.1 μg of inactivated vaccine could inhibit the immunization of offspring with 30 μg of HA DNA vaccine. The survival rates of offspring were only 50% and 43% respectively (Table 3). The inactivated vaccine at high doses showed the inhibition to the offspring immunization with HA DNA. The result was similar to the report by Radu et al. [6]. Whereas, the maternal antibody induced by 0.01 μg of inactivated vaccine showed no inhibition to the offspring immunization with HA DNA. The survival rate of offspring after a lethal virus challenge reached 86% (6/7). On the contrary, when both female mice and their offspring were immunized with 0.01 μg of inactivated vaccine, no offspring survived the lethal virus challenge. It showed no significant inhibition of maternal antibody induced by low dose inactivated vaccine to the immunization of offspring with HA DNA.

Though the maternal immunization with inactivated vaccine partially inhibited the offspring immunization with HA DNA, it did not inhibit the offspring immunization with NA DNA. As shown in Table 3, the anti-NA antibody titers of offspring immunized with NA DNA were only slightly lower than those of offspring born to the unimmunized mothers, and the offspring were completely protected from a lethal virus challenge. The dose of inactivated vaccine showed no influence on the immunization of NA DNA in offspring. This might be due to the low NA protein content in inactivated vaccine, which induced a low titer of maternal anti-NA antibody. So the immunization of female mice with inactivated vaccine and their offspring with NA DNA was a good immune strategy to overcome the interference of maternal antibody.

Another immune strategy, i.e. the immunization of mother and the offspring with DNA vaccines encoding different antigens also showed a satisfactory result in our study. As shown in Table 4, the offspring immunized as schemed were completely protected. The anti-HA antibody titers of HA DNA-immunized offspring, born to the mothers immunized with NA DNA, were comparable to those of HA DNA-immunized offspring born to unimmunized mothers. Moreover, the protective rate of the former was significantly higher than that of the latter, which might be due to the enhancement of protective effect by maternal anti-NA antibody. Similarly, the anti-NA antibody titers in NA DNA-immunized offspring, born to the mothers immunized with HA DNA, were comparable to that of NA DNA-immunized offspring born to unimmunized mothers.

In this study the NA antibody titers were determined by fluorescent-based NI assay with the substrate 2'-(4-methylumbeliferyl)-α-D-N-acetylneuraminic acid (4-MU). We notice there is a report showing that the assay may be unreliable and lack standardization compared with the fetuin-based assay [31]. However, we don't think it make a big difference to draw valid conclusions in this study. Although the derived antibody titer may not be accurate because of the limitation of the NI assay, it would not influence the general changing tendency of all the antibody titers and thus would not influence the conclusions. Moreover, our conclusions are based on other factors, which we think are more important, such as survival rate and residual lung virus titer of mice, etc. The NA antibody titer determined by fluorescent-based NI assay would be further confirmed by fetuin-based assay in our future studies.

## Conclusion

In this study, we designed the above experiments to explore the influence of maternal antibody on offspring and tried to find ways to overcome its inhibition effect. All the experiments were performed twice. From the results, we may conclude that, in order to avoid the interference of maternal antibody with the vaccination of offspring, the immune strategy for mothers and their offspring shall be adjusted as follows: 1) If mothers are immunized with inactivated influenza vaccine, the offspring shall be immunized with NA DNA vaccine, and the immunization with inactivated vaccine shall be avoided in offspring; 2) If mothers are immunized with influenza virus HA (NA) DNA, the offspring shall be immunized with NA (HA) DNA, and the immunization with the same DNA vaccine as that for mothers shall be avoided as far as possible.

## Methods

### Plasmid DNAs

Plasmids pCAGGSP7/NA and pCAGGSP7/HA were constructed by cloning the PCR products of NA and HA genes from the influenza virus strain A/PR/8/34 (PR8, H1N1) into expression vector pCAGGSP7, respectively, as described previously [32]. Plasmid pCAGGSP7 was constructed by inserting a polylinker (oligonucleotides) containing *Kpn*I, *Xho*I, *Cla*I, *EcoR*V, *Sma*I, *Not*I and *Sac*I sites into the *EcoR*I site of pCAGGS which was constructed by Niwa *et al*. [33]. The nucleotide sequences of NA and HA DNAs were confirmed by the dideoxy method using ABI PRISM 377 DNA Sequencer (Applied Biosystems). The expressions of the encoded proteins, NA and HA, were also confirmed in 293T human embryonic kidney cells, as described previously [32]. Plasmids were propagated in *Escherichia coli *XL1-blue bacteria and purified using QIAGEN purification kits (QIAGEN-tip 500, Qiagen, Chatsworth, CA).

### Immunization

The female BALB/c mice in test groups were immunized twice, at an interval of 3 weeks, with the vaccine at various dosages respectively. One week after the second immunization, the female mice were bred with unimmunized male BALB/c mice aged 10–12 weeks in the same cage. About 15 days later, most of the females became pregnant and were then separated from the males. The offspring were grouped, 11–12 for each, and used for the subsequent test. Offspring of unimmunized mothers were produced by the same procedure.

The inactivated split-product influenza virus A/PR/8/34 (PR8, H1N1) vaccine was diluted to a volume of 200 μl with phosphate buffer saline (PBS). Adult female mice (aged 6–8 weeks) or neonatal mice (aged 1 week) were immunized i.p. with 1.0, 0.1 and 0.01 μg of inactivated influenza vaccine separately. Three weeks later, the mice were boosted with the vaccine at same dose as primed. Inactivated split-product influenza virus (PR8, H1N1) vaccine was prepared by Shanghai Institute of Biological Products (SIBP) and detected for concentration using BCA kit (Pierce).

In vivo electroporation was carried out according to the method described by Aihara and Miyazaki [34]. Adult female BALB/c mice (aged 6 to 8 weeks) and neonatal mice (aged 1 week) were immunized twice with plasmid DNA dissolved in 30 and 15 μl of Tris-EDTA buffer, respectively. After injection in the right quadriceps muscle, a pair of electrode needles with 5 mm apart was inserted into the muscle to cover the DNA injection sites and electric pulses were delivered using an electric pulse generator (Electro Square Porator T830 M; BTX, San Diego, CA). Three pulses of 100 V each, followed by three pulses of the opposite polarity, were delivered to each injection site at a rate of one pulse per second. Each pulse lasted for 50 ms.

### Infection

One week after the second immunization, the offspring were anesthetized and challenged with 20 μl of viral suspension of mouse-adapted strain A/PR/8/34 (PR8, H1N1) [20 × 50% lethal dose (LD_50_)] by intranasal route. This infection caused rapid and widespread viral replication in the lungs and deaths of the unimmunized mice within 7 days [35].

### Specimens

At least three mice in each group were anaesthetized with chloroform and then bled from the heart with a syringe. After bleeding, the mice were incised ventrally along the median line from the xiphoid process to the point of the chin. The trachea and lungs were taken out and washed three times by injecting with a total of 2 ml of PBS containing 0.1% BSA. The bronchoalveolar wash was used for virus titration after removing cellular debris by centrifugation [36].

### Ab assays

The sera were collected by tail vein bleeding and used for IgG Abs assays. The titer of IgG Ab produced against HA molecules purified from PR8 virus was measured by enzyme-linked immunosorbent assay (ELISA). ELISA was performed using a 96-well microtiter plate (EIA plate, Costar, Cambridge, MA, USA) with the reagents consisting of first, HA molecules purified from the PR8 virus according to the procedure described by Phelan *et al *[37]; second, 2-fold serial dilutions of sera from each group of immunized or unimmunized mice; third, goat anti-mouse IgG Ab (γ-chain specific) (Southern Biotechnology Associates, Inc. USA) conjugated with biotin; fourth, streptavidine conjugated with alkaline phosphatase (Southern Biotechnology Associates, Inc. USA); and finally, *p*-nitrophenyl-phosphate. The amount of chromogen produced was measured based on absorbance at 414 nm and 405 nm in a Labsystems Multiskan Ascent Autoreader (model 354, Finland).

The inhibition assay of NA activity by Ab (NI assay) was performed with the substrate 2'-(4-methylumbeliferyl)-α-D-N-acetylneuraminic acid (4-MU). PR8 viruses (10^7 ^EID_50 _per ml), grown in the allantoic sac of 10-day-old chicken embryos and stored as allantoic fluid suspensions at -80°C, were employed as enzyme source. NI assay was carried out by preincubating the enzymes (25 μl) with various dilutions of antiserum (25 μl) at room temperature for 30 min before the enzyme assay. The enzyme mixtures were then incubated with the substrate solution (50 μl) containing 400 mmol/L sodium acetate (pH 6.5), 4 mmol/L calcium chloride and 2% butanol in the presence of 1 mmol/L 4-MU. After incubation at 37°C for 10–30 min, the reaction was stopped by adding a mixture (100 μl) containing 665 mmol/L glycine, 415 mmol/L sodium hydrogen carbonate and 300 mmol/L sodium chloride (pH 10.7). Levels of free 4-MU were measured by reading the fluorescence intensity on a microtiter plate fluorescence reader (Genios, Tecan) at 365 nm. Antibody-positive cut-off values were set as the mean-2 × S.D. of the fluorescence intensity of mice in control group. NI titers are expressed as the highest serum dilution that resulted in inhibition of the NA activity [38-40].

### Virus titrations

The bronchoalveolar wash was diluted 10-fold serially starting from a dilution of 1:10, inoculated to Madin-Darby canine kidney (MDCK) cells, incubated at 37°C and examined for cytopathic effect 3 days later. The virus titer of each specimen, expressed as the fifty percent tissue culture infection dose (TCID_50_), was calculated by Reed-Muench method [41]. The virus titer in each experimental group is represented by the mean ± S.D. of the virus titers per ml of specimens from at least 3 mice in each group.

### Statistics

The results of experimental groups were evaluated by Student's *t*-test; if *P *value is less than 0.05, the difference was considered as significant. For survival, the probability was calculated by using Fisher's exact test, comparing the survival rate of mice immunized with vaccine to that of the mice in control groups. The survival patterns of the control and the immunized mice were graphed using the Kaplan-Maier survival curves. The Log Rank test was used to analyze the survival rate data. Differences were considered significant at *p *< 0.05.

## Authors' contributions

JJC, FHZ and FF did most of the experimental work. HYC participated in the analysis of antibody. ZC conceived of the study, and participated in its design and coordination. All authors read and approved the final manuscript.

## Pre-publication history

The pre-publication history for this paper can be accessed here:


